# Nasal immunization with AMH-INH-RFRP DNA vaccine for improving follicle development and fertility in buffaloes

**DOI:** 10.3389/fendo.2023.1076404

**Published:** 2023-02-20

**Authors:** Chao Chen, Xuhong Zhao, Zhigao An, Muhammad Jamil Ahmad, Kaifeng Niu, Xinxin Zhang, Pei Nie, Jiaomei Tang, Aixin Liang, Liguo Yang

**Affiliations:** ^1^ National Center for International Research on Animal Genetics, Breeding and Reproduction (NCIRAGBR), College of Animal Science and Technology, Huazhong Agricultural University, Wuhan, China; ^2^ Faculty of Veterinary and Animal Sciences, Muhammad Nawaz Shareef University of Agriculture, Multan, Pakistan; ^3^ College of Veterinary Medicine, Northwest Agricultural and Forestry University, Yangling, China; ^4^ Hubei Engineering Research Center in Buffalo Breeding and Products, Wuhan, China

**Keywords:** AMH, INH, RFRP, DNA vaccine, reproduction, buffalo

## Abstract

**Introduction:**

Inhibin DNA vaccine has already been proven to improve the fertility of animals. This study aimed to investigate the effects of a novel Anti-Müllerian hormone (AMH)-Inhibin (INH)-RF-amide-related peptides (RFRP) DNA vaccine on immune response and reproductive performance in buffalo.

**Methods:**

A total of 84 buffaloes were randomly divided into four groups and nasally immunized twice a day with 10 ml of either AMH-INH-RFRP DNA vaccines (3 × 10^10^ CFU/ml in group T1, 3 × 10^9^ CFU/ml in group T2, and 3 × 10^8^ CFU/ml in group T3) or PBS (as a control) for 3 days, respectively. All animals received a booster dose at an interval of 14 days.

**Results:**

ELISA assay revealed that primary and booster immunization significantly increased the anti-AMH, anti-INH, and anti-RFRP antibody titers in the T2 group compared with that in the T3 group. After the primary immunization, the antibody positive rate was significantly higher in the T2 group than that in the T3 group. In addition, ELISA results indicated that concentrations of E2, IFN-γ, and IL-4 were significantly higher in the antibody-positive (P) group compared to the antibody-negative (N) group. In contrast, there was no significant difference in the concentrations of P4 between the P and N groups. Ultrasonography results revealed a highly significant increase of 2.02 mm in the diameter of ovulatory follicles in the P group compared to the N group. In parallel, growth speed of dominant follicles was significantly higher in the P group than that in the N group (1.33 ± 1.30 vs 1.13 ± 0.12). Furthermore, compared to N group, the rates of oestrus, ovulation, and conception were also significantly higher in the P group.

**Conclusion:**

The novel AMH-INH-RFRP DNA vaccine improves the proportion of oestrus, ovulation, and conception in buffalo by promoting the production of E2 and the growth of follicles.

## 1 Introduction

Buffalo has important economic and biological significance for tropical and subtropical regions due to characteristics of good adaptability and stress resistance (1). Compared with cattle, buffaloes have many known reproductive disorders including poor sign of estrus, longer postpartum quiescence, delayed puberty, and seasonal breeder, resulting in the reduction of buffalo fertility and the buffalo industry’s developmental limits (2–4). Biotechnologies such as estrus synchronization and fixed-timed artificial insemination (FTAI) have been well known for improving the fertility of buffalo (5). Nevertheless, some researchers reported that the conception rate was approximately 30% of buffaloes using Ovsynch-TAI protocol (6, 7), suggesting that the reproductive effect induced by Ovsynch-TAI protocol is not always satisfactory. The main reason might be due to differences in the age and diameter of the preovulatory follicles during synchronized ovulation with the second GnRH treatment, resulting in a different level of maturation of the ovulatory follicles (8).

Inhibin (INH) has long been thought to be a hormone that inhibits follicle-stimulating hormone (FSH) secretion and regulates ovarian function through pituitary-gonadal negative feedback (9, 10). Notably, the recombinant inhibin DNA vaccine delivered by Salmonella choleraesuis has already been proven to improve the fertility of mice, rats, and sheep (11–13). Meanwhile, Liu Q et al. reported that the proportion of estrous and ovulation could be improved by immunization with recombinant inhibin DNA vaccine, eventually leading to an increase in the conception rate of buffaloes (14, 15). A novel hypothalamic neuropeptide called gonadotropin-inhibitory hormone (GnIH) is known to inhibit FSH and luteinizing hormone (LH) from the anterior pituitary since its detection in quail. In mammals, it is also called RF-amide-related peptides (RFRP), which have molecules similar to GnIH (16, 17). In addition, the applying a recombinant DNA vaccine harboring the INH and RFRP genes has revealed better immunogenicity and more litter size of mice (18). Anti-Müllerian hormone (AMH), a member of the transforming growth factor-beta (TGF-β) super-family, is responsible for the regression of the Müllerian duct during sexual differentiation in males (19). In females, AMH has been shown to suppress the primordial follicle initiation, and inhibit FSH-dependent increase in aromatase activity, LH receptor expression, and COC *in vitro* maturation (20–22). Moreover, it inhibited the growth of mouse secondary follicles (23). Yu X et al. reported that co-immunization with AMH and INH plasmids could increase the litter size of mice (24).

Based on the above studies, it can be concluded that INH, INH-RFRP, and INH-AMH DNA vaccines exhibit promising effects on enhancing animals’ reproductive performance. Here, the current study aimed to investigate the immune response of a novel AMH-INH-RFRP DNA vaccine harboring AMH, INH, and RFRP encoding genes, and evaluate the reproductive effect of this novel vaccine on oestrus, ovulation, and conception in buffalo.

## 2 Materials and methods

### 2.1 Preparation of AMH-INH-RFRP DNA vaccine

**Figure 1:**
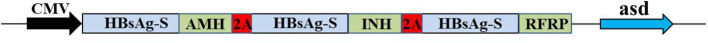
Schematic map for the construction of AMH-INH-RFRP DNA vaccine.

AMH-INH-RFRP DNA vaccine has previously been constructed and preserved in our laboratory. Briefly, the recombinant plasmid AMH-INH-RFRP contained three fused fragments including S/AMH, S/INH, and S/RFRP, which were linked with 2A peptide (**Figure 1**). Subsequently, AMH-INH-RFRP plasmid was transformed into attenuated salmonella choleraesuis (C500) with asd and crp double deletion, producing AMH-INH-RFRP DNA vaccine delivered by C500 strain.

AMH-INH-RFRP DNA vaccine was added (1: 100) to LB liquid medium (200 ml) and cultured in 37°C shaker at 220 rpm/min for 16 h. After harvesting through 4°C centrifugation at 6000 rpm for 10 min, the vaccine was re-suspended by phosphate-buffered saline (PBS). Before immunization, LB agar plates were used to place the bacteria in triplicate to determine the number of colony-forming units and acquire adjusted roughly 3 × 10^10^ CFU/ml by PBS.

### 2.2 Animals and immunization protocol

Experimental buffaloes (n = 84) of 3-6 years old with first to third lactations were from local buffalo farms (Hubei JinNiu Co., Ltd. Shayang, China) and enrolled in this study, which has the body condition score (BCS) between 2.5 to 3 (1-5 score) and are free from reproductive problems. Experimental animals were fed a total mixed ration (TMR) containing forages and concentrates in routine. Forages were comprised of corn silage, rice straw, peanut, and concentrates included corn (38.0%), soybean meal (16.0%), linen (6.0%), cottonseed cake (6.0%), cornmeal (17.5%), vinasse (10.0%), baking soda (0.5%) and premixed material (6.0%). The experimental animals had free access to fresh and clean water during the experimental period. A guideline followed for using animals and experimental procedures was from the Animal Care and Use Committee of Huazhong Agricultural University (HZAUBU-2019-001).

**Figure 2:**
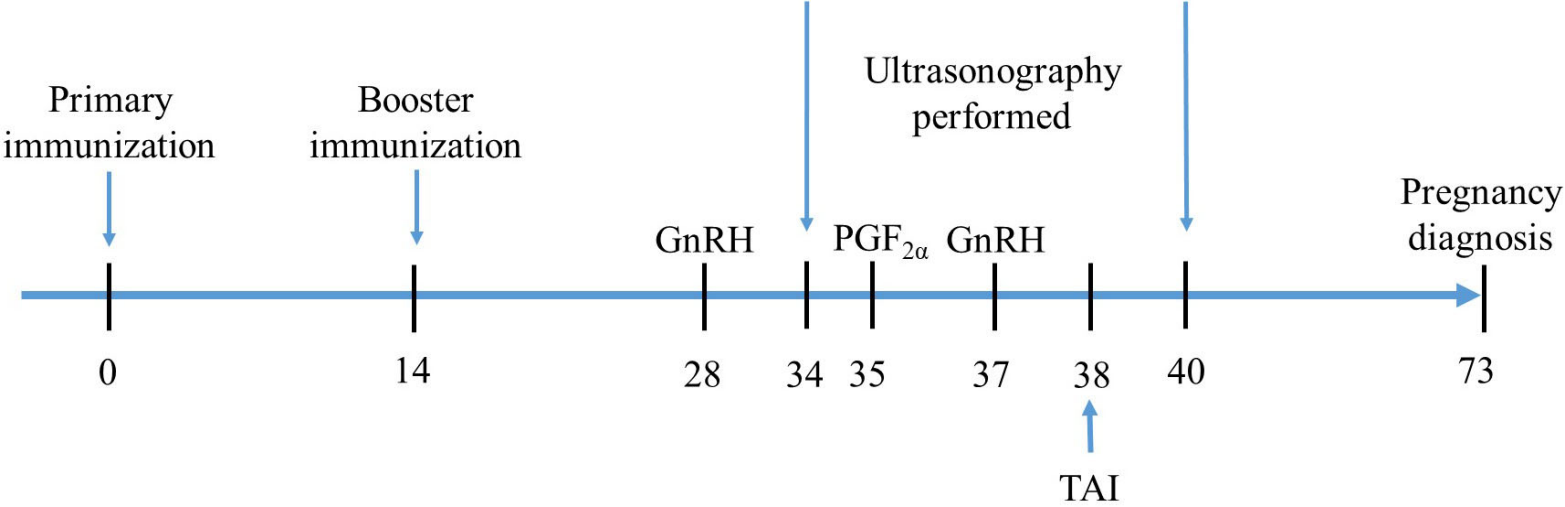
An outline of experimental design. Experimental buffaloes were immunized with AMH-INH-RFRP DNA vaccine on days 0 and 14, and control group were administered with PBS. All buffaloes underwent the Ovsynch-TAI program after 2 weeks of booster immunization. Ultrasonography was conducted from day 34 to 40 at 12 h interval to evaluate the folliculogenesis and ovulation. At day 73, pregnancy diagnosis was performed for all buffaloes.

A total of 84 buffaloes was randomly divided into four groups: groups T1 (3 × 10^10^ CFU/ml, n = 21), T2 (3 × 10^9^ CFU/ml, n = 21), and T3 (3 × 10^8^ CFU/ml, n = 21) were nasally immunized twice a day with 10 ml of AMH-INH-RFRP DNA vaccine for 3 days, respectively. The control group (n = 21) was nasally immunized twice daily with 10 ml PBS for 3 days. At 2 weeks after primary immunization, all animals were boosted with the same procedures as primary immunization. After a booster immunization, buffaloes were subjected to the classic Ovsynch-TAI protocol (GPG). During the experiment, a total of 10 buffaloes did not participate in the complete GPG procedure due to buffalo injuries and management negligence. The details of the treatment procedure are presented in **Figure 2**. During the experiment, blood samples from the jugular vein of buffaloes were collected on days 14 and 28. The blood was then centrifuged at 3000 rpm for 10 min to separate the serum. The serum samples were stored at -80°C for further analysis.

### 2.3 Ultrasonography detection

From day 34 (a day earlier than PGF2α treatment) to day 40 (72 h post 2^nd^ GnRH injection), all buffaloes were scanned using B-type ultrasound (Shenzhen Well.D Medical Electronics Co., Ltd. China) to evaluate follicles and ovulation at 12 h intervals. Oestrus was observed and confirmed by obvious vaginal mucous discharge twice a day (8:00 and 16:00). Ovulation was confirmed at the subsequent ultrasonographic examination by the abrupt disappearance of the previously recorded dominant follicle. At 24 h intervals, the prominent follicles were compared to determine their growth rate (mm/day). A pregnancy diagnosis was conducted on the 35th day following artificial insemination (AI) to assess the rate of conception.

### 2.4 Oestrous synchronization procedure

The Ovsynch-TAI program was used in this study. Briefly, all buffaloes were injected with 200 μg of GnRH analog (Sansheng Pharmaceutical Co., Ltd., Ningbo, China) on day 28. Further, after an intramuscular injection of 0.4 mg PGF2α (Sansheng Pharmaceutical Co., Ltd., Ningbo, China), the buffaloes received another shot of 200 μg GnRH on day 37. From 18 to 24 h after the second GnRH injection, TAI was performed for all buffaloes.

### 2.5 Detection of antibody titer

Anti-AMH, INH, and RFRP antibodies in serum were measured by indirect ELISA. Briefly, each 96-well microtiter plate was coated 100 ng/100 μl AMH, INH, and RFRP antigens, which were synthesized by Sangon-Peptide Biotech CO., Ltd. (Shanghai, China), and then incubated overnight at 4°C. After discarding the reaction reagent, 300 μl of PBS with Tween 20 (Tween 20, 1247ML100, Bioroxx, Germany) (PBST) was added into each well and washed for 5 times. Next, 200 μl of 1% bovine serum albumin (BSA, 4240GR100, Bioroxx, Germany) was added into each well for blocking and then incubated at 37°C for 90 min. After washing with PBST, serum samples (100 μl) diluted with PBS (1:25, 1:50, 1:100, 1:200, 1:400, 1:800, 1:1600, and 1:3200) were added into each well and incubated at 37°C for 1 h. Following PBST wash, goat anti-bovine IgG-HRP (1:5000; 6030-05, Southern Biotechnology Associates, Inc. USA) was added (100 μl/well) and incubated at 37°C for 1 h. After washing with PBST, 150 μl of tetramethylbenzidine (TMB, ATO00001, AtaGenix, Wuhan, China) were added into each well and incubated at 37°C for 15 min. Then 50 μl of stop solution (C1058, Solarbio Science & Technology Co., Ltd. China) were added to stop the reaction. Finally, the absorbance at 450 nm was measured by a Microplate Reader (PerkinElmer, USA). Under the same dilution factor, if the OD450 value of the experimental group was greater than the mean OD450 value in the control group + 2SD (standard deviation), it was judged as a positive value (18, 25).

### 2.6 Assay of reproductive hormone and cytokine concentrations

Bovine ELISA Kits (Ruixin Biological Technology Co., Ltd.; Quanzhou, China) were used to measure concentrations of IL-4 (RX1600854B), IFN-γ (RX1600863B), E2 (RXJ1600845B), and P4 (RXJ1600748B) concentrations, according to the manufacturer’s instructions. The intra- and inter-assay coefficients of variation were less than 10.0% and 15.0% for IL-4, 15.0% and 15.0% for IFN-γ, 15.0% and 15.0% for E2, and 15.0% and 15.0% for P4, respectively.

### 2.7 Statistical analyses

The data were analysed using the MIXED procedure of SAS 9.4 software (SAS Institute, Inc., Cary, NC, USA). The mean values were compared using Tukey’ multiple range test with a significance level of p < 0.05. Nonparametric data were analysed using the chisquared test. Variability in the data was expressed as the standard error of means (SEM), and p < 0.05 was considered statistically significant.

## 3 Results

### 3.1 Immune response of AMH-INH-RFRP DNA vaccine against buffalo

**Figure 3:**
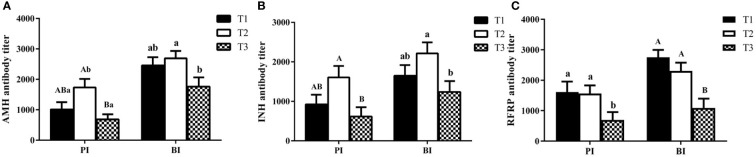
The titers of IgG antibody in buffaloes after immunization with various concentration of AMH-INH-RFRP DNA vaccine. Anti-AMH **(A)**, anti-INH **(B)**, anti-RFRP **(C)** were detected by ELISA. Data are presented as mean ± SEM. The bars with different letters indicate significant differences (^a,b^ *P* < 0.05, ^A,B^ *P* < 0.01).

As shown in **Figure 3**, buffaloes immunized with AMH-INH-RFRP DNA vaccine could induce anti-AMH, anti-INH, and anti-RFRP antibodies on day 14 after primary immunization (PI) and booster immunization (BI). After primary immunization, T2 group showed remarkably higher anti-AMH and anti-INH antibody levels than T3 group (*P* < 0.01), and T1 and T2 groups exhibited significantly higher anti-RFRP antibody titer than T3 group (*P* < 0.05). After booster immunization, there was an increasing trend in anti-AMH, anti-INH, and anti-RFRP antibody titers. Anti-AMH and anti-INH antibody titers in T2 group were significantly greater than that in T3 group (*P* < 0.05), and anti-RFRP antibody titers were significantly greater in T1 and T2 groups compared to that in T3 group (*P* < 0.01).

On the other hand, we also calculated the antibody positive rate in different treatment groups (**Table 1**). After primary immunization, the anti-AMH antibody positive rate in T1 and T2 groups was significantly higher than that in T3 group (85.71% and 85.71% vs 47.62%, *P* < 0.01). In addition, the antibody positive rates for anti-INH, and anti-RFRP in T2 group were significantly increased by 38.09% compared to T3 group (*P* < 0.05). After booster immunization, the antibody positive rates increased compared to primary immunization, even though there were no significant changes among different groups.

**Table 1:** Percentage (and proportion) of buffalo with positive antibody against AMH, RFRP and INH at various intervals after immunization of DNA vaccine.

Group	Number	Primary immunization			Booster immunization		
		AMH	RFRP	INH	AMH	RFRP	INH
T1	21	85.71 (18/21)^a^	76.19 (16/21)^ab^	76.19 (16/21)^ab^	95.24 (20/21)	95.24 (20/21)	90.48 (19/21)
T2	21	85.71 (18/21)^a^	85.71 (18/21)^a^	85.71 (18/21)^a^	95.24 (20/21)	100.00 (21/21)	95.24 (20/21)
T3	21	47.62 (10/21)^b^	47.62 (10/21)^b^	47.62 (10/21)^b^	76.19 (16/21)	80.95 (17/21)	90.48 (19/21)
P value		0.0058	0.0199	0.0199	0.0764	0.0594	0.8047

Different superscripts in the same column indicate a significant difference between various groups (^a,b^P < 0.05).

### 3.2 The effect of AMH-INH-RFRP DNA vaccine on steroid hormones and cytokines in buffalo

**Figure 4:**
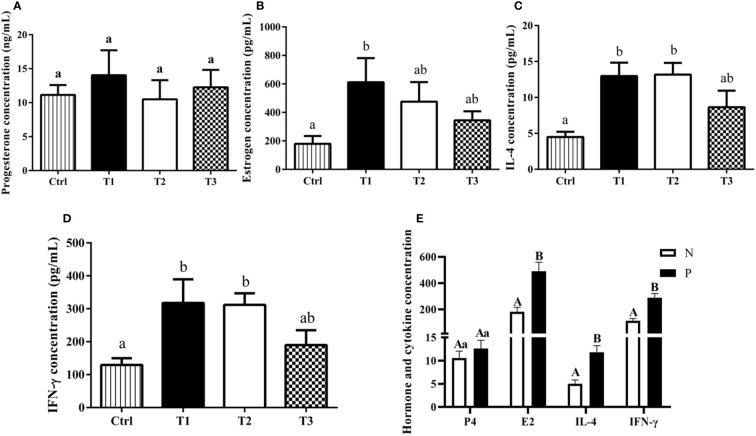
The effects of AMH-INH-RFRP DNA vaccine on steroid hormones and cytokines in buffalo after nasal immunization. **(A-D)** represent the effect on P4, E2, IFN-γ, and IL-4, respectively. **(E)** The effect of antibody-positive and antibody-negative buffaloes on P4, E2, IL-4 and IFN-γ. Data are presented as mean ± SEM. The bars with difference letters indicated significant differences (^a,b^ *P* < 0.05; ^A,B^ *P* < 0.01).

The levels of steroid hormones (P4 and E2) and cytokines (IL-4 and IFN-γ) were detected by ELISA kits (**Figure 4**). Regarding serum P4 concentration, no significant difference was observed between the different groups (*P* > 0.05, **Figure 4A**). Compared with the control (Ctrl) group, the E2 concentration in T1 group significantly increased by 432.65 pg/mL (*P* < 0.05, **Figure 4B**). In addition, we observed that the levels of IL-4 and IFN-γ were significantly greater in T1 and T2 groups than that in Ctrl group (*P* < 0.05, **Figures 4C, D**). Furthermore, we compared the differences in steroid hormones and cytokines between the antibody-positive (P) and antibody-negative (N) buffaloes. The results showed that the concentrations of IL-4, IFN-γ and E2 in antibody-positive buffaloes were significantly increased compared to those in antibody-negative buffaloes (*P* < 0.01), while there was no significant difference in the concentration of P4 (*P* > 0.05, **Figure 4E**).

### 3.3 The effect of AMH-INH-RFRP DNA vaccine on folliculogenesis in buffalo

All buffaloes were monitored by ultrasonography to record the diameter of follicle and the growth rate of the dominant follicle (mm/day). As shown in **Table 2**, the buffaloes in T1 (12.98 ± 0.46), T2 (13.55 ± 0.44) and T3 (12.56 ± 0.46) groups had a significantly greater diameter of ovulatory follicle compared to the Ctrl group (10.74 ± 0.46, *P* < 0.05). In addition, there was a significant increase in the growth speed of dominant follicle in T1 (1.34 ± 0.07) and T2 (1.32 ± 0.08) groups compared to Ctrl group (1.10 ± 0.08, *P* < 0.05). However, there were no significant differences in diameter of ovulatory follicles and the growth speed of dominant follicle between DNA vaccine immunization groups (*P* > 0.05).

**Table 2:** The diameter of ovulatory follicles and dominant follicles’ growth speed in buffaloes after immunization with different vaccine concentrations.

Groups	Diameter of ovulatory follicle (mm)	Growth speed of dominant follicle (mm/day)
Ctrl	10.74 ± 0.46^a^	1.10 ± 0.08^a^
T1	12.98 ± 0.46^b^	1.34 ± 0.07^b^
T2	13.55 ± 0.44^b^	1.32 ± 0.08^b^
T3	12.56 ± 0.46^b^	1.28 ± 0.08^ab^

Different superscripts in the same column indicate a significant difference between various groups (^a,b^P < 0.05). Data are indicated mean ± SEM.

Moreover, we further compared the changes in diameter of ovulatory follicles and the growth speed of dominant follicle between antibody-positive and antibody-negative buffaloes. As shown in **Table 3**, the diameter of ovulatory follicles in the P group was highly significantly increased by 2.02 mm compared to the N group (*P* < 0.01), and growth speed of dominant follicle in the P group was much faster than that in the N group (1.33 ± 0.04 vs 1.13 ± 0.06, *P* < 0.05).

**Table 3:** Diameter of ovulatory follicles and growth speed of dominant follicle between antibody-positive and antibody-negative buffaloes.

Groups	Diameter of ovulatory follicle (mm)	Growth speed of dominant follicle (mm/day)
N	11.19 ± 0.34^a^	1.13 ± 0.06^a^
P	13.21 ± 0.28^b^	1.33 ± 0.04^b^

Different superscripts in the same column indicate a significant difference between various groups (^a,b^P < 0.05). Data are indicated as mean ± SEM.

### 3.4 The effects of AMH-INH-RFRP DNA vaccine on oestrus, ovulation, and conception rates

After immunization with AMH-INH-RFRP DNA vaccine, the oestrus, ovulation, and conception rates of buffaloes were observed and recorded. As shown in **Table 4**, oestrus and conception rates of buffaloes in groups T1, T2, and T3 tended to be higher than the Ctrl group, although the difference didn’t reach significance (*P* > 0.05). Furthermore, the proportion of ovulation in T1, T2, and T3 groups was significantly superior to that of the Ctrl group by 35.67%, 36.84%, and 35.67%, respectively (*P* < 0.05).

**Table 4:** Rates of oestrus, ovulation, and conception between different groups.

Variable	Treatment groups				P-value
	T1	T2	T3	Ctrl	
Oestrus (%)	61.11 (11/18)	63.16 (12/19)	61.11 (11/18)	36.84 (7/19)	0.31
Ovulation (%)	77.78^a^ (14/18)	78.95^a^ (15/19)	77.78^a^ (14/18)	42.11^b^ (8/19)	0.03
Conception (%)	38.89 (7/18)	36.84 (7/19)	33.33 (6/18)	26.32 (5/19)	0.85

Different superscripts in the same row indicate a significant difference between various groups (^a,b^P < 0.05).

Notably, there were extremely significant differences in oestrus and ovulation rates between antibody-positive and antibody-negative buffaloes in **Table 5** (*P* < 0.05). Likewise, the conception rate in P group was also significantly higher by 24.03% than that in N group (*P* < 0.05).

**Table 5:** Oestrus, ovulation and conception rates between antibody-positive and antibody-negative buffaloes.

Variable	P	N	P-value
Oestrus (%)	65.96^a^ (31/47)	29.63^b^ (10/27)	0.02
Ovulation (%)	78.72^a^ (38/47)	44.44^b^ (13/27)	0.00
Conception (%)	42.55^a^ (20/47)	18.52^b^ (5/27)	0.04

Different superscripts in the same row indicate a significant difference between various groups (^a,b^P < 0.05).

## 4 Discussion

It is well known that DNA vaccine has several obvious advantages over conventional vaccines, including easy to design and manufacture, low cost, convenient transportation, unsafe sources of infection are not involved, and encoding multiple immunogenic epitopes (26). Unfortunately, inadequate immunogenicity is still the biggest challenge for application of practical DNA vaccines (27). In the present study, to improve the immune response of DNA vaccine, the hepatitis B surface antigen gene (HBsAg-S) was used to fuse with AMH, INH, and RFRP genes, and 2A peptide was employed to ligate the fragments of S/AMH, S/INH, and S/RFRP (28, 29). DNA vaccines can induce both humoral and cellular immune responses (30). Our results showed that antibody titers were higher in T1 and T2 groups than that in T3 group after both primary and booster immunizations. There was no significant difference between T1 and T2 groups, suggesting that the antibody levels evoked by AMH-INH-RFRP DNA vaccines are not in a dose-dependent manner. Notably, all experimental groups showed a high rate of antibody positivity after 14 days of booster immunization, ensuring booster efficacy. On the other hand, we further measured expression levels of IL-4 and IFN-γ, and observed that IL-4 and IFN-γ were significantly higher in the P group than that in the N group. T helper (Th) cells play a key role in regulating the immune response by synthesizing and releasing cytokines into the surrounding microenvironment. The Th1 cells produce IL-2, IFN-γ, tumor necrosis factor (TNF), and lymphotoxin and are associated with cell-mediated immune functions. The Th2 cells produce IL-4, IL-5, IL-6, and IL-10, to assist the humoral immune response (31, 32). Inagawa et al. found that IFN-γ and IL-4 significantly enhanced specific Th1 and Th2 cell immune responses, respectively (33). All the above evidence suggests that AMH-INH-RFRP DNA vaccine can induce both humoral and cellular immune response through nasal immunization in buffalo, which is consistent with the previous studies on GPV-VP1 DNA vaccine (34).

It was previously reported that the concentrations of E2 were significantly higher in large follicles compared to those in medium and small follicles, but the progesterone concentrations were not affected in buffaloes (35). In addition, E2 levels in plasma correlated with estrus performance in buffalo (36). Estrogens can promote granulosa cell proliferation and follicle growth and inhibit atresia of small luminal follicles or preluminal follicles, and also promote maturation of large follicles and ovulation (37). Importantly, cumulative studies showed that INH showed an additive inhibitory effect on FSH-induced estradiol production (10, 24). Similarly, AMH and RFRP also can negatively regulate the synthesis and secretion of steroid hormones by downregulation the aromatase (38, 39). Here, we found that antibody-positive buffaloes had higher serum estrogen levels than antibody-negative buffaloes, the possible reason may be due to the decrease of endogenous AMH, INH, and RRPP hormones, which were immune neutralized by anti-AMH, anti-INH, and anti-RFRP antibodies (13, 15). However, the immunized and control groups did not show any change in P4 concentration, presumably since mature follicles did not ovulate. Corpus luteum (CL) formation didn’t occur (40). In addition, the increase of estrogen in antibody-positive buffaloes significantly accelerates the growth speed of the dominant follicle, and increase the diameter of the ovulatory follicle, results in high proportions of estrus and ovulation. Agree with Wang et al. studies in rats, showing that plasma concentration of FSH and E2 was significantly increased following pcISI DNA vaccination in rats and stimulated follicular development (41).

A previous study demonstrated that estrus synchronization in buffalo with Ovsynch resulted in the estrus rate of 96.1%, ovulation of 84.6%, and pregnancy rate of 30.8% (5), which are higher than our control result. The possible reason may be attributed to the season of experiment which was between May and July in this study, which is the low breeding season for buffalo (6). Evidence showed that buffaloes had a greater calving incidence and oestrous expression from August to November in most parts of India (42, 43). Moreover, pregnancy rates were poor during the low breeding season showing that 26% in cyclic buffalo and 7% in acyclic buffalo synchronized with Ovsynch (7, 44). On the other hand, sexed semen for TAI in this study might be another important reason for the low conception rate. Though sexed semen is attributed to improving the genetic merit of future cows, however, the conception rate with sexed semen is low (45). In a previous study, compared to the conventional semen group (55%), cattle in the sexed semen group (44%) had an 11% reduction in conception rate (46). Although conception rates in N group were low due to seasonal and sexed semen factors, by a combination of AMH-INH-RFRP DNA vaccine immunization and Ovsynch-TAI protocol, we observed that the conception rate in T1, T2, T3 groups increased to 38.89%, 36.84%, and 33.33% as compared to Ctrl group (26.32%), and the conception rate in the P group was significantly increased by 24.03% compared to the N group (42.55% vs 18.52%). This data indicated that AMH-INH-RFRP DNA vaccine could overcome the problem of poor effects resulting from Ovsynch-TAI protocol in non-breeding seasons and the use of sexed semen.

## 5 Conclusions

In summary, nasal immunization against buffalo with the AMH-INH-RFRP DNA vaccine induced the cellular and humoral immune responses, and increased the estrogen concentration. Meanwhile, AMH-INH-RFRP DNA vaccine increased the diameter of ovulatory follicle and accelerated the growth speed of dominant follicle, thus improving the behavioral oestrus and ovulation, ultimately leading to an increase in the conception rate of buffaloes.

## Data availability statement

The raw data supporting the conclusions of this article will be made available by the authors, without undue reservation.

## Ethics statement

The animal study was reviewed and approved by Animal Care and Use Committee of Huazhong Agricultural University (HZAUBU-2019-001). Written informed consent was obtained from the owners for the participation of their animals in this study.

## Author contributions

CC performed the whole experiment and wrote the manuscript. CC and MA conceived and designed the experiment data curation. ZA, PN, XXZ, KN, JT, and XHZ contributed animals’ arrangement and sample collection. AL and LY supervised the study and revised the manuscript. All the authors have reviewed the manuscript. All authors contributed to the article and approved the submitted version.
